# Giant spin-torque diode sensitivity in the absence of bias magnetic field

**DOI:** 10.1038/ncomms11259

**Published:** 2016-04-07

**Authors:** Bin Fang, Mario Carpentieri, Xiaojie Hao, Hongwen Jiang, Jordan A. Katine, Ilya N. Krivorotov, Berthold Ocker, Juergen Langer, Kang L. Wang, Baoshun Zhang, Bruno Azzerboni, Pedram Khalili Amiri, Giovanni Finocchio, Zhongming Zeng

**Affiliations:** 1 Key Laboratory of Nanodevices and Applications, Suzhou Institute of Nano-tech and Nano-bionics, Chinese Academy of Sciences, Ruoshui Road 398, Suzhou 215123, China; 2 Department of Electrical and Information Engineering, Polytechnic of Bari, Bari 70125, Italy; 3 Department of Physics and Astronomy, University of California, Los Angeles, California 90095, USA; 4 HGST Inc, 3403 Yerba Buena Road, San Jose, California 95135, USA; 5 Department of Physics and Astronomy, University of California, Irvine, California 92697, USA; 6 Singulus Technologies, Kahl am Main 63796, Germany; 7 Department of Electrical Engineering, University of California, Los Angeles, California 90095, USA; 8 Department of Engineering, University of Messina, Messina 98166, Italy; 9 Department of Mathematical and Computer Sciences, Physical Sciences and Earth Sciences, University of Messina, Messina 98166, Italy

## Abstract

Microwave detectors based on the spin-torque diode effect are among the key emerging spintronic devices. By utilizing the spin of electrons in addition to charge, they have the potential to overcome the theoretical performance limits of their semiconductor (Schottky) counterparts. However, so far, practical implementations of spin-diode microwave detectors have been limited by the necessity to apply a magnetic field. Here, we demonstrate nanoscale magnetic tunnel junction microwave detectors, exhibiting high-detection sensitivity of 75,400 mV mW^−1^ at room temperature without any external bias fields, and for low-input power (micro-Watts or lower). This sensitivity is significantly larger than both state-of-the-art Schottky diode detectors and existing spintronic diodes. Micromagnetic simulations and measurements reveal the essential role of injection locking to achieve this sensitivity performance. This mechanism may provide a pathway to enable further performance improvement of spin-torque diode microwave detectors.

## Abstract (web summary)

Nanomagnetic materials allow for the emission and detection of microwave radiation in technological applications by spin excitation. Here, the authors present sensitive room-temperature microwave detectors based on nanoscale magnetic tunnel junctions, enabled via spin torque and injection locking.

## Graphical abstract

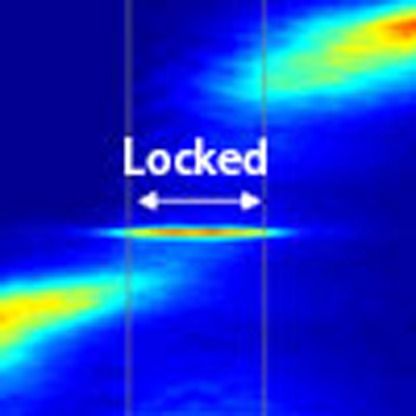

The continuous progress in the development of magnetic materials and nanostructures has enabled spintronic devices with performance superior to semiconductor-based electronics, offering promising solutions for a range of future high-speed and energy-saving electronic systems^1^,^2^,^3^,^4^,^5^,^6^,^7^. In particular, the spin-transfer torque^8^,^9^ induced by d.c. spin-polarized current can switch the magnetization^10^,^11^, or excite self-oscillations^12^,^13^ giving rise to applications such as memories and nanoscale oscillators^14^,^15^,^16^. On the other hand, microwave detectors (rectifiers) on the basis of the spin-torque diode effect can be realized when the d.c. input is replaced by a microwave current^17^,^18^. The spin-torque diode effect is the result of spin-torque-induced ferromagnetic resonance (FMR), which leads to a rectification effect (that is, d.c. voltage, *V*_dc_) in magneto-resistive nanoscale devices. Since its discovery^17^, this effect has been used for quantitative measurements of magnetic torques in magnetic tunnel junctions (MTJs) (for example, Slonczewski, field-like, and voltage-controlled torques)^19^,^20^,^21^,^22^.

For practical applications in microwave detectors, the ability to obtain a device with high-detection sensitivity without using external magnetic fields, at room temperature and at low-input microwave powers is crucial. However, there are currently no practical (spintronic or conventional semiconductor) solutions that achieve all of these requirements simultaneously. In spin-torque diodes studied so far, the application of an additional external magnetic field (often canted at an angle with respect to the device plane) is required to achieve large microwave detection sensitivity^21^,^23^,^24^,^25^,^26^,^27^,^28^. Although this external field may in principle be integrated into the device, for example, by engineering the material stack, or by using coils or permanent magnets, it is undesirable from a practical point of view due to increased size and cost of the device. On the other hand, semiconductor-based Schottky diode detectors, while not requiring a magnetic bias, fail to offer sufficient sensitivity for low input microwave powers due to the thermodynamic limit (the theoretical limit being ∼4,000 mV mW^−1^)^28^.

In this work, we present a spin-torque diode microwave detector meeting all of the above-mentioned requirements. This is achieved by incorporating three elements into the device. First, a perpendicularly magnetized free layer^6^,^7^,^29^, which allows for device operation in the absence of external magnetic fields^30^. Second, an MgO-based MTJ material stack exhibiting high-tunnel magnetoresistance (TMR). Third, the injection-locking mechanism^31^ due to the simultaneous application of d.c. and microwave currents. The operation mechanism and the fundamental role of the injection locking are discussed on the basis of measurements combined with micromagnetic simulations.

## Results

### Spin-torque diode device

**Figure 1:**
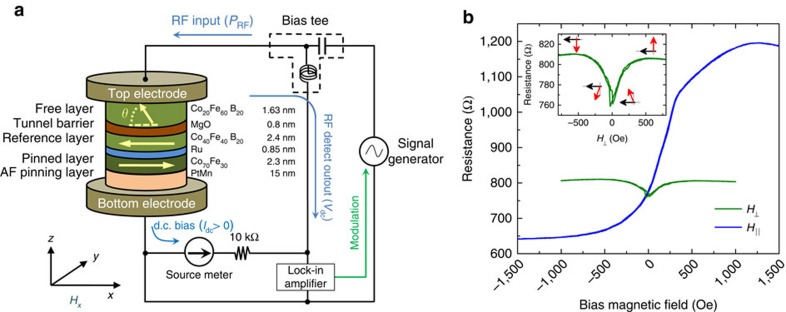
Spin-torque diode device. (**a**) Spin-torque diode device and schematic of circuit used for FMR measurements. The device is based on an MTJ with an in-plane magnetized reference layer and a perpendicularly magnetized free layer, separated by an MgO tunnel barrier. The detected voltage (*V*_dc_) is measured by a low-frequency (10 kHz) modulation method using a lock-in amplifier. (**b**) The magnetoresistance curve of the MTJ device under in-plane magnetic field (*H*_‖_) and perpendicular magnetic field (*H*_⊥_) for d.c. current (*I*_dc_) of 10 μA. The resistance scan as a function of the out-of-plane field (inset of **b**) clearly indicates the perpendicular free layer. The black (red) arrow denotes the magnetization direction of the reference (free) layer.

The devices studied in this work have an MTJ structure consisting of a synthetic antiferromagnetic Co_70_Fe_30_ (2.3 nm)/Ru (0.85 nm)/Co_40_Fe_40_B_20_ (2.4 nm) reference layer, exchange biased by a PtMn film, designed to have an in-plane easy axis, and a Co_20_Fe_60_B_20_ perpendicularly magnetized free layer, separated from the reference layer by a 0.8 nm MgO tunnel barrier. A schematic of the device is shown in Fig. 1a. The free layer has an out-of-plane easy axis at zero bias field, which is realized by controlling the perpendicular magnetic anisotropy at the interface of the CoFeB layer with the MgO tunnel barrier^6^,^7^,^29^,^30^. This magnetization configuration enables the device to excite large-amplitude magnetization precession under a small spin-torque^30^. In addition, the CoFeB–MgO–CoFeB material combination ensures high TMR^4^,^5^,^6^,^7^. These factors are key ingredients to enhance the sensitivity of the spin-torque diode. Electron-beam lithography and ion milling were used to fabricate the pillar-shaped devices with elliptical cross-section. All data in the main text are from one 150 nm × 60 nm device. Other measured samples exhibited similar behaviour.

Figure 1b shows the resistance as a function of the in-plane magnetic field applied parallel to the ellipse major axis (*H*_‖_), at a bias current of *I*_dc_=10 μA. As the field increases from −1,000 to +1,000 Oe, the resistance increases gradually as the free-layer magnetization aligns anti-parallel to the reference layer magnetization. The resistance curve scan as a function of the out-of-plane field (*H*_⊥_) (inset of Fig. 1b) clearly indicates the perpendicular free layer. At zero field, a small tilting angle (*θ*=76°) from the out-of-plane configuration is measured due to the coupling between the free and reference layers. We estimated *θ*, the angle between the magnetization vectors of the free layer and the reference layer, from the MTJ resistance^28^


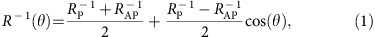


where the resistances in the anti-parallel (*R*_AP_) and parallel (*R*_P_) configurations are 1,200 and 640 Ω, respectively. In addition, the free layer exhibits a voltage-controlled interfacial perpendicular magnetic anisotropy (VCMA) estimated to be 34 fJV^−1^m^−1^ (see Supplementary Fig. 1 and Supplementary Note 1), in good agreement with previous reports for similar material structures^21^,^22^,^32^,^33^. All measurements reported below were carried out at room temperature and zero bias magnetic field.

### Detection properties

**Figure 2:**
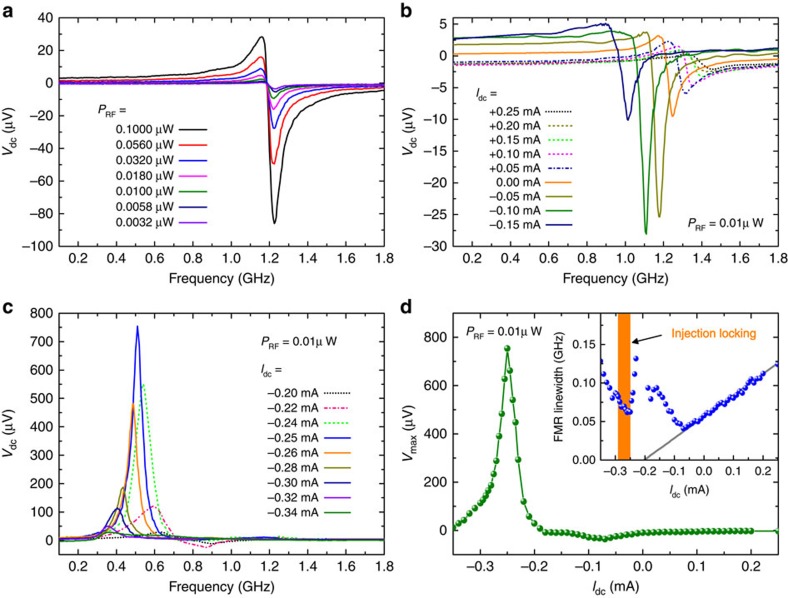
Voltage detection characteristics of spin-torque diode device. (**a**) Detection voltage (*V*_dc_) as a function of microwave frequency, for various input microwave powers (*P*_RF_) at zero d.c. bias current. (**b**,**c**) *V*_dc_ as a function of microwave frequency under various d.c. bias currents (*I*_dc_). The d.c. bias was found to significantly affect *V*_dc_. (**d**) Maximum *V*_dc_ as a function of the d.c. bias. The inset in **d** shows the spectral linewidth of the free-layer FMR as a function of *I*_dc_. By extrapolating the fitting line (grey solid line), the critical current (*I*_c_) for magnetization stability in the free layer is estimated to be −0.2 mA.

We studied the spin-torque diode response by using FMR measurements as shown in Fig. 1a (ref. 18). A weak microwave current *I*_ac_ sin (2*πf*_ac_*t*) and a d.c. current *I*_dc_ were applied to the device through a bias Tee using a signal generator (E8257D, Agilent Technologies) and a source metre (2400, Keithley). At *I*_dc_=0 mA, when a microwave current at a frequency *f*_ac_ is applied, the free layer magnetization starts to precess at the same frequency, resulting in a time-dependent resistance oscillation due to the TMR effect. As a result, a rectified voltage is generated across the MTJ^17^. To improve the signal-to-noise ratio, the microwave input was modulated at a low frequency (10 kHz), and the resulting rectified voltage *V*_dc_ was measured with a lock-in amplifier (SR830, Standard Research Systems). Figure 2a shows the measured rectified voltage as a function of the microwave frequency at *I*_dc_=0 mA, for an input microwave power ranging from 3.2 to 100 nW. The maximum voltage is measured at the resonant frequency *f*_0_=1.2 GHz. The FMR spectra in Fig. 2a are well fitted by a sum of symmetric and antisymmetric Lorentzian functions with identical resonant frequency *f*_0_. The origin of the asymmetric line shape is related to the VCMA effect^21^. The detection sensitivity, defined as the rectified voltage divided by the incident microwave power (*P*_RF_), that is, *V*_dc_/*P*_RF_, was obtained to be 970 mV mW^−1^ (see Supplementary Fig. 2 for similar data from an additional device). This is on the same level as previously reported values (440 and 630 mV mW^−1^)^21^,^28^ for MTJ-based diode detectors, and at a comparable level to unbiased commercial Schottky diode detectors (500–1,000 mV mW^−1^, Herotek, Inc.).

We next conducted spin-torque diode response studies under different d.c. bias currents. Figure 2b,c shows the detected voltage curves as a function of the microwave frequency, at a low-input microwave power of 10 nW, for the range of d.c. bias currents from −0.34 to +0.25 mA. Positive d.c. currents were found to suppress the detection voltage, because in this case spin torque increases the damping of the magnetization precession, while VCMA increases the perpendicular anisotropy in the free layer. For a range of negative currents (−0.32<*I*_dc_ < −0.22 mA), the detection voltages were significantly enhanced and the spectra shifted to a lower frequency. Figure 2d summarizes the maximum rectified voltage (*V*_max_) as a function of d.c. bias current, while the inset of Fig. 2d shows the spectral linewidth of the spin-torque FMR data, with the indication of the region where the larger detection voltage is measured. As discussed later in detail, this corresponds to the current region where the injection locking is achieved.

**Figure 3:**
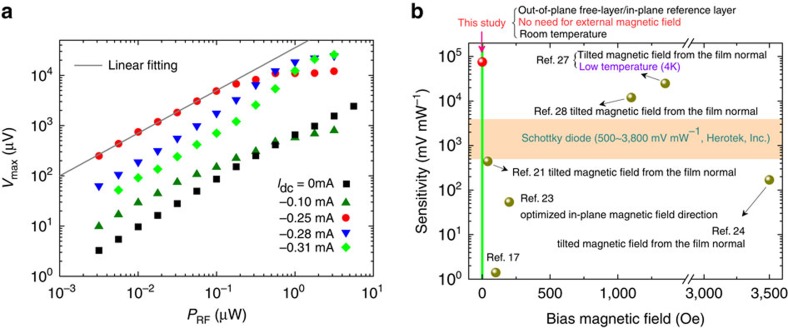
RF power dependence and the comparison of detection sensitivity with the indication of the required bias field. (**a**) The dependence of output voltage on the RF input power under different d.c. bias current (*I*_dc_) values. (**b**) The detection sensitivity values reported previously require an external magnetic field with a particular direction and amplitude, while in this study the giant sensitivity is achieved in the absence of any bias magnetic field.

Figure 3a shows the detection voltage as a function of *P*_RF_ for various d.c. bias currents. The device shows a quadratic detection dependence on the d.c. bias, consistent with a previous study^28^. Moreover, a large detection voltage (∼mV order) is achieved for small input power. For example, the maximum detected voltage reaches 754 μV at *I*_dc_=−0.25 mA and *P*_RF_=0.01 μW, which is about 80 × larger than the one measured at zero bias current. The corresponding detection sensitivity of 75,400 mV mW^−1^ is substantially larger than state-of-the-art biased Schottky diode detectors (3,800 mV mW^−1^, Herotek, Inc.) and the best existing spintronic diodes (12,000 mV mW^−1^), which additionally require large magnetic bias fields for their operation^28^. Figure 3b compares our results with previously reported detection sensitivities for different spin-torque diodes, with the indication of the required external magnetic field. It can be observed that the devices reported in this work represent the combination of high-detection sensitivity and bias-field-free operation. In addition, the device exhibits signal-to-noise ratio, which is of the same order as those of previous reports^28^ (see Supplementary Note 2 and Supplementary Figs. 3,4,5). It is worth noting that the giant detection sensitivities observed here are not only substantially larger than those of Schottky diodes, but they are also achieved at low microwave input power (<100 nW). By comparison, Schottky diodes typically do not provide satisfying microwave-to-d.c. conversion efficiency for sub-μW input microwave power^34^. Furthermore, the spin-torque diode devices can be scaled down to nano-metre size (0.07 μm^2^ in this study), which makes them potentially suitable for compact on-chip microwave detectors.

### Discussion of physical mechanism

We next focus on the mechanisms responsible for the large-voltage detection sensitivity. Under d.c. bias, two previous experiments demonstrated that the detection voltage can be enhanced due to the nonlinear FMR^27^,^28^. In thermally assisted nonlinear FMR^27^ (or stochastic resonance), an applied radiofrequency (RF) current excites a large-amplitude precession with the assistance of thermal energy, and the detection voltage shows an exponential dependence on the RF input power. Another type of nonlinear FMR (called nonlinear FMR with asymmetric potential), in which the large detection voltage is linked to a rotation of the orbital centre of the free-layer magnetization, has been also observed^28^. The maximum detection voltage is achieved near *I*_dc_=*I*_c_, where *I*_c_ is the critical current at which the equilibrium configuration of the magnetization is destabilized by the spin-transfer torque^28^. By comparison, the device discussed in this article, exhibits quadratic detection properties for small RF input power (see Fig. 3a), and enhanced detection voltage in the specific range from −0.22 to −0.32 mA (see Fig. 2d), which is above *I*_c_. Here, *I*_c_ is estimated to be −0.2 mA, from the extrapolation of the fitted line in the d.c. current dependence of the FMR spectral linewidths, as shown in the inset of Fig. 2d. Hence, the underlying physics in this study is different from that of nonlinear FMR observed in previous experiments.

An alternative mechanism is the theory related to the out-of-plane precession^35^, in which the device operates as a non-resonant broadband microwave detector, and the output voltage is virtually independent of the input microwave power. The device discussed here exhibits a resonant character (see Fig. 2b,c), and the output voltage depends on *P*_RF_ (see Fig. 3a). Hence, the mechanism driving the large detection sensitivity in this study is different from this picture as well.

### Role of injection locking on detection behaviour

**Figure 4:**
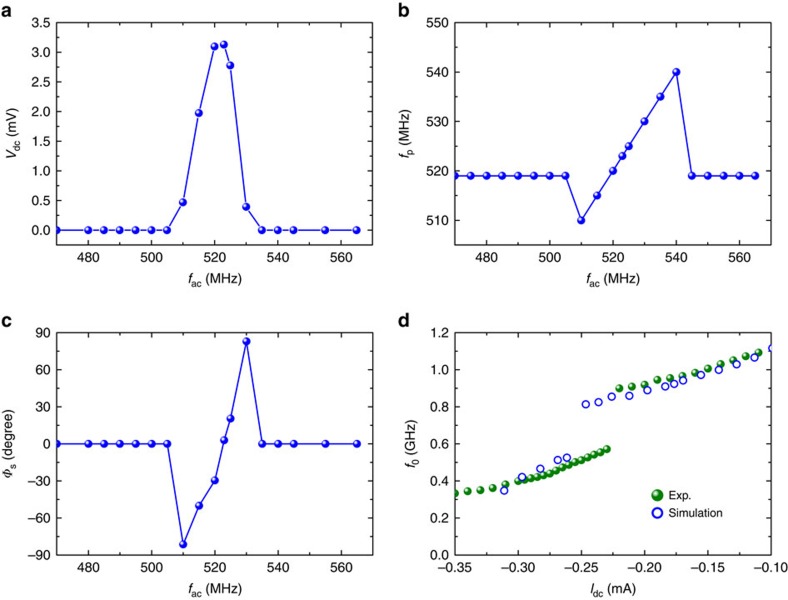
Micromagnetic simulations. (**a**) Rectified voltage and (**b**) Oscillation frequency (*f*_p_) of the microwave emission, as a function of the input microwave frequency (*f*_ac_). (**c**) Intrinsic phase shift between the microwave current and the oscillating TMR signal. The results of micromagnetic simulations are obtained for d.c. bias currents *I*_dc_=−0.26 mA and microwave current *I*_ac_=7 μA. (**d**) A comparison between the experimental and simulated resonant frequency (*f*_0_) of the maximum detection voltage as a function of *I*_dc_.

To gain a deeper understanding of the ultrahigh sensitivity mechanism, we performed micromagnetic simulations (see Methods). Our computational results show persistent magnetization dynamics (self-oscillations) (at *I*_ac_=0 mA), as well as injection locking (at *I*_ac_>0 mA)^31^,^36^,^37^,^38^. Figure 4a shows an example of the detection voltage as a function of frequency (*f*_ac_), as computed by means of micromagnetic simulations for *I*_dc_=−0.26 mA and *I*_ac_=7 μA (*P*_RF_=0.15 μW). The large detection voltage is observed in the frequency range corresponding to the locking region (between 510 and 540 MHz), as shown in Fig. 4b, where it can be clearly observed that the microwave emission frequency *f*_p_ is locked to the frequency *f*_ac_ of the input microwave current. Within the locking region, an intrinsic phase shift *Φ*_s_ (ref. 39) exists between the oscillation resistance and the microwave current. The origin of *Φ*_s_ can be understood from the theory developed in ref. 40, in which it is directly linked to the coupling between the amplitude and the frequency of the self-oscillation (see equation (56) in ref. 40). It is noted that the value of *Φ*_s_ inside the locking bandwidth depends on the microwave frequency (Fig. 4c), similar to that in a previous study^41^. Figure 4d shows a comparison between the experimental and theoretical values for the frequency *f*_0_ of the maximum rectification voltage, as a function of the d.c. bias current. The simulation data (open circles) are quantitatively consistent with the experimental data (olive colour), namely, a red shift is observed in the frequency *f*_0_ with increasing the amplitude of the negative current, along with a frequency jump of about 300 MHz. This analysis points to a scenario where the injection locking is responsible for the observed giant detection sensitivity.

**Figure 5:**
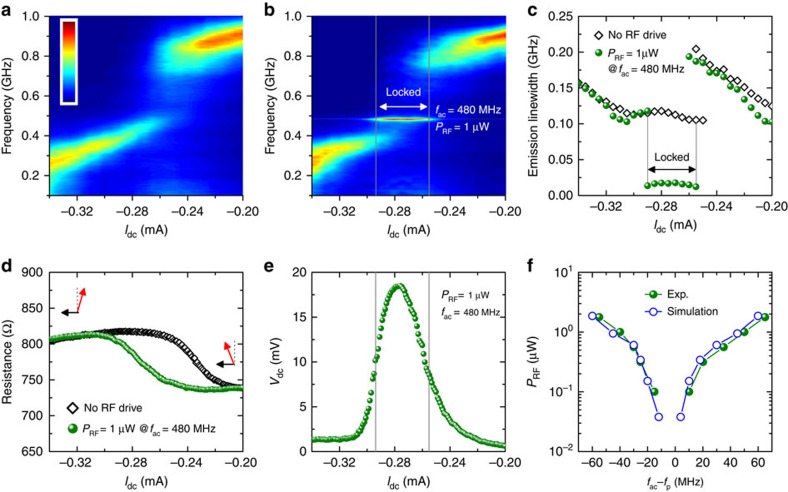
Injection locking of spin-torque microwave emission to an external microwave signal. (**a**) Plot of spin-torque microwave emission spectra as a function of d.c. bias currents *I*_dc_, with amplitude shown in a linear colour scale from 0 (blue) to 40 nW GHz^−1^ (red). (**b**) Same as **a** but with a RF drive at 480 MHz and RF power *P*_RF_=1 μW. (**c**) Spectral linewidth of microwave emission as a function of *I*_dc_, with/without a RF drive deduced from **a** and **b**. (**d**) Device resistance as a function of *I*_dc_ with and without the RF drive. The precession axis of the free-layer magnetization (red arrows) and reference layer polarization (black arrows) are also indicated. (**e**) The detected voltage *V*_dc_ as a function of *I*_dc_. Vertical lines indicate the region of locking. (**f**) A comparison between experimental values (olive dots) and micromagnetic calculations (blue circles) of the locking range as a function of RF drive amplitude, at *I*_dc_=−0.26 mA.

To further verify the role of the injection locking, we conducted additional experiments to study the current-induced microwave emission^12^ with and without a RF input in our samples, recorded using a 9 kHz–26.5 GHz spectrum analyzer (see Supplementary Fig. 6). The output of microwave emissions in the absence of *P*_RF_ as a function of *I*_dc_ is shown in Fig. 5a. The oscillation frequency (*f*_p_) exhibits a red shift with increasing the bias current. The frequency jump in the *f*_p_ versus *I*_dc_ response is related to the sign change of the *x*-component of the oscillation axis from negative to positive, as also indicated from the change in the resistance curve (see Fig. 5d). Similar behaviour was also observed in spin-torque oscillators at low out-of-plane bias fields^42^. Near *I*_dc_=−0.34 mA, the steady magnetization dynamics are switched off, analogous to the disappearance of the detection spectrum in the spin-torque diode measurements (see Fig. 2c). Figure 5b shows the analogous data when an RF drive *P*_RF_=1 μW at 480 MHz is added to *I*_dc_. At *I*_dc_=−0.25 mA the oscillation frequency locks to the drive frequency, and the locking continues up to *I*_dc_=−0.29 mA. At larger bias currents the oscillation frequency is unlocked from the drive frequency. In the locking range, the linewidth of the microwave emission is significantly narrowed by a factor of 8 × (to 15 MHz), as can be observed in Fig. 5c, but is still much larger than the linewidth of the external microwave signal (on the order of several Hertz). These results indicate that the frequency-locking does not always result in a full phase-locking of the spin-torque nano-oscillator to the external microwave signal, consistent with previous reports^37^,^38^. This can be attributed to the influence of noise^37^,^38^,^43^, that is, the external microwave signal has to compete with noise, which results in fluctuations of the oscillator phase.

Despite full phase-locking not being reached, the detection voltage *V*_dc_ is drastically increased as shown in Fig. 5e. Quantitatively, for a fixed frequency, the detection voltage is given by 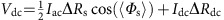 (refs 27, 28), where Δ*R*_dc_(*I*_ac_)=*R*_dc_(*I*_ac_)−*R*_dc_(0) is the difference between the average resistance in the presence and in the absence of *I*_ac_, while 〈*Φ*_s_〉 is the time-average intrinsic phase shift, and Δ*R*_s_ is the amplitude of the oscillating resistance in the presence of both *I*_dc_ and *I*_ac_. It is worth pointing out that the value of Δ*R*_s_ can be significantly larger in the presence of d.c. bias, given that it results from the amplitude of the self-oscillation of the magnetization, rather than conventional FMR. As an example, for *I*_dc_=−0.26 mA and *I*_ac_=18 μA (*P*_RF_=1 μW, *f*_ac_=480 MHz), from Fig. 5d Δ*R*_dc_(*I*_ac_)=60 Ω, while from the output power (Fig. 5b) it can be estimated that the oscillation is due to 30% of the total TMR signal, which corresponds to Δ*R*_s_=170 Ω. Considering 〈*Φ*_s_〉≈0, the calculated detection voltage of ∼17.13 mV is consistent with the measurements in Fig. 5e. From a theoretical point of view, according to the above analysis the detection voltage is limited by the maximum TMR signal. In Fig. 5f, the locking range is more explicitly shown as a function of the RF drive amplitude. The locking range is deduced from the dependence of the oscillation frequency *f*_p_ on the drive frequency *f*_ac_, where the microwave emission is locked to the injected signal (see Fig. 4b for simulation results, or Supplementary Note 3 and Supplementary Fig. 7 for additional experimental data). The locking range increases with the drive amplitude, which is consistent with the simulation data and in agreement with the analytical theory^40^.

## Discussion

In summary, we have demonstrated giant sensitivity of nanoscale spintronic diodes in the absence of any external magnetic field, at room temperature and for low-input microwave power. The analysis of microwave emission measurements with and without RF input, as well as micromagnetic simulations, reveal that injection locking is the key mechanism to achieve this large sensitivity. It is anticipated that this mechanism may provide a pathway for development of high-performance spintronic microwave detectors.

## Methods

### Sample preparation

The continuous multilayer thin films with stacks of composition PtMn (15)/Co_70_Fe_30_ (2.3)/Ru (0.85)/Co_40_Fe_40_B_20_ (2.4)/MgO (0.8)/Co_20_Fe_60_B_20_ (1.63) (thickness in nm) were deposited using a Singulus TIMARIS physical vapour deposition system and annealed at 300 °C for 2 h in a magnetic field of 1 T. The films were subsequently patterned into ellipse-shaped pillars using optical and electron-beam lithography combined with ion milling. The resistance-area product in the parallel magnetization configuration was 4.5 Ω μm^2^, and the in-plane TMR ratio, defined as (*R*_AP_−*R*_P_)/*R*_P_, was 87.5%.

### Micromagnetic simulations

We numerically solve the Landau–Lifshitz–Gilbert–Slonczewski equation which includes the field-like torque *T*_OP_ (refs 44, 45), and the voltage dependence of the anisotropy, that is, VCMA^21^. The *T*_OP_ is considered to be dependent on the square of the bias voltage up to a maximum value of 10% of the in-plane torque, computed for a current density |*J*|=4.0 × 10^6 ^A cm^−2^. (ref. 19). However, our simulations show that the field-like torque term does not qualitatively influence the detection voltage, as already observed in the scenario of ref. 21. The total torque, including also the in-plane component *T*_IP_ is given by


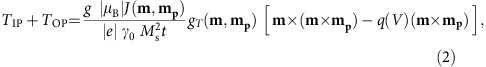


where *g* is the gyromagnetic splitting factor, *γ*_0_ is the gyromagnetic ratio, *μ*_B_ is the Bohr magneton, *q*(*V*) is the voltage-dependent parameter for the perpendicular torque, *J*(**m**,**m**_**p**_) is the spatially nonuniform current density, *V* is the voltage (computed from *I*_dc_−*R* curves, see Fig. 5d), *t* is the thickness of the free layer, and *e* is the electron charge. The effective field takes into account the standard micromagnetic contributions (exchange, self-magnetostatic) as well as the Oersted field due to both microwave and d.c. currents. The presence of the VCMA has been modelled as an additional contribution to the effective field. The parameters used for the CoFeB are saturation magnetization *M*_s_=9.5 × 10^5^ A m^−1^, exchange constant *A*=2.0 × 10^−11^ J m^−1^, and damping parameter *α*=0.02. The zero bias anisotropy constant *k*_u_=5.52 × 10^5^ J m^−3^ has been estimated from the fitting of the FMR frequency *f*_0_=1.245 GHz from Fig. 2a, while the VCMA coefficient is 34 fJV^−1^m^−1^ (see Supplementary Note 1). The minimum value of the *k*_u_ achieved at *I*_dc_=−0.35 mA is 5.45 × 10^5^ J m^−3^. The polarization function *g*_*T*_(**m**, **m**_**p**_)=2*η*_*T*_(1+2*η*_*T*_^2^ **m**·**m**_**p**_)^−1^, where **m** and **m**_**p**_ are the normalized magnetizations of the free and pinned layers, has been computed by Slonczewski^46^,^47^. We have used for the spin-polarization *η*_*T*_ the value 0.6 (ref. 19).

## Additional information

**How to cite this article**: Fang, B. *et al*. Giant spin-torque diode sensitivity in the absence of bias magnetic field. *Nat. Commun.* 7:11259 doi: 10.1038/ncomms11259 (2016).

## Supplementary Material

Supplementary Information Supplementary Figures 1-7, Supplementary Notes 1-3 and Supplementary References
