# Supplementary material for: Giant spin-torque diode sensitivity in the absence of bias magnetic field
Source: Nat Commun. 2016 Apr 7;7:11259. doi: 10.1038/ncomms11259 (PMC4829691; doi:10.1038/ncomms11259)
Supplement: Supplementary Information — Supplementary Figures 1-7, Supplementary Notes 1-3 and Supplementary References [file ncomms11259-s1.pdf]

Supplementary Information

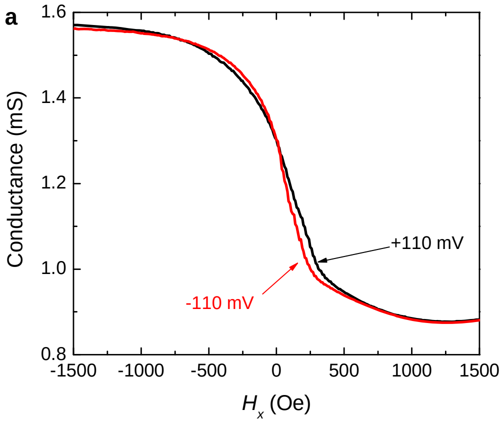

Supplementary Figure 1: Effect of the voltage on the perpendicular magnetic anisotropy. (**a**) Hysteresis loop of device conductance (G) versus in-plane magnetic field (H_x) measured under ±110 mV direct voltage bias V_{dc} for the device (Device #1) presented in the main text. (**b**) Variation of the effective perpendicular anisotropy field $\Delta H_p(V_{dc})$ with d.c. voltage, extracted from the $G(H_x)$ hysteresis loops via Supplementary Equation (1) in Supplementary Note 1 (black squares), and a linear fit to the data (red line).

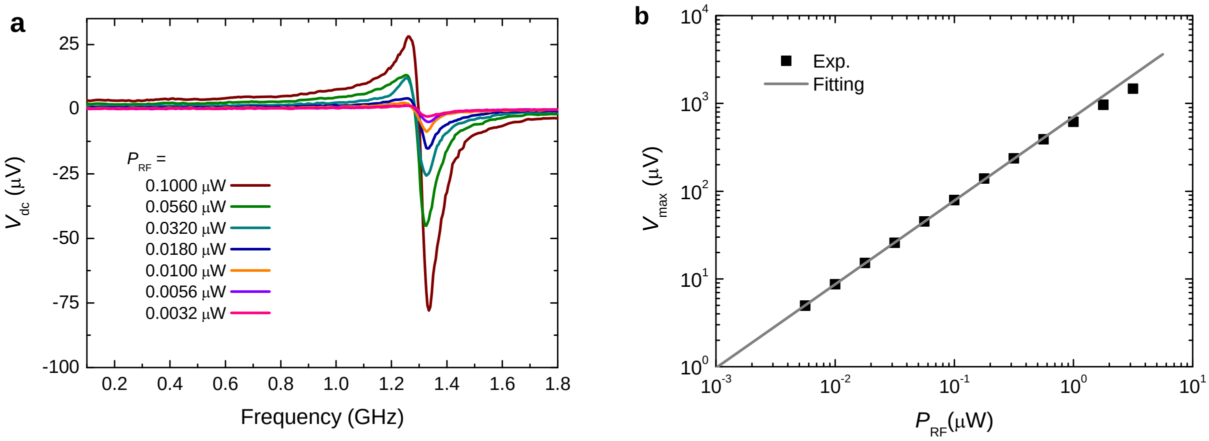

Supplementary Figure 2: Voltage detection characteristics of one 150 nm×50 nm device (Device #2). (**a**) Detection voltage (V_{dc}) as a function of microwave frequency for various input microwave powers (P_{RF}) at zero d.c. bias current. (**b**) The maximum detection voltage (V_{max}, taken from the peak voltage values in panel **a**), as a function of P_{RF} in the absence of d.c. bias current.

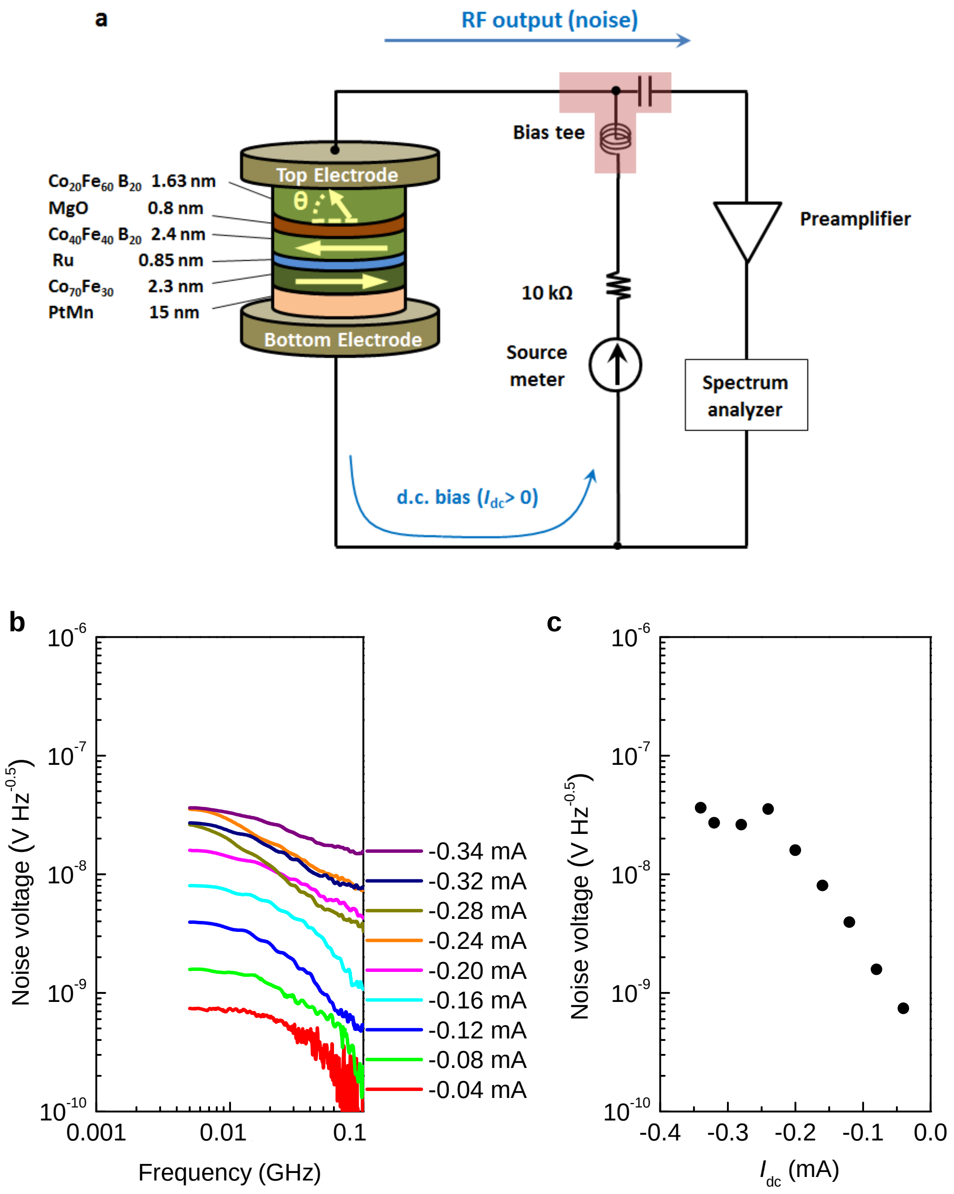

Supplementary Figure 3: Nonlinear magnetic noise. (**a**) The measurement circuit for non-linear magnetic noise. (**b**) Noise voltage versus frequency curves for various d.c. bias currents. (**c**) d.c. bias (I_{dc}) dependence of the nonlinear magnetic noise around 0 Hz.

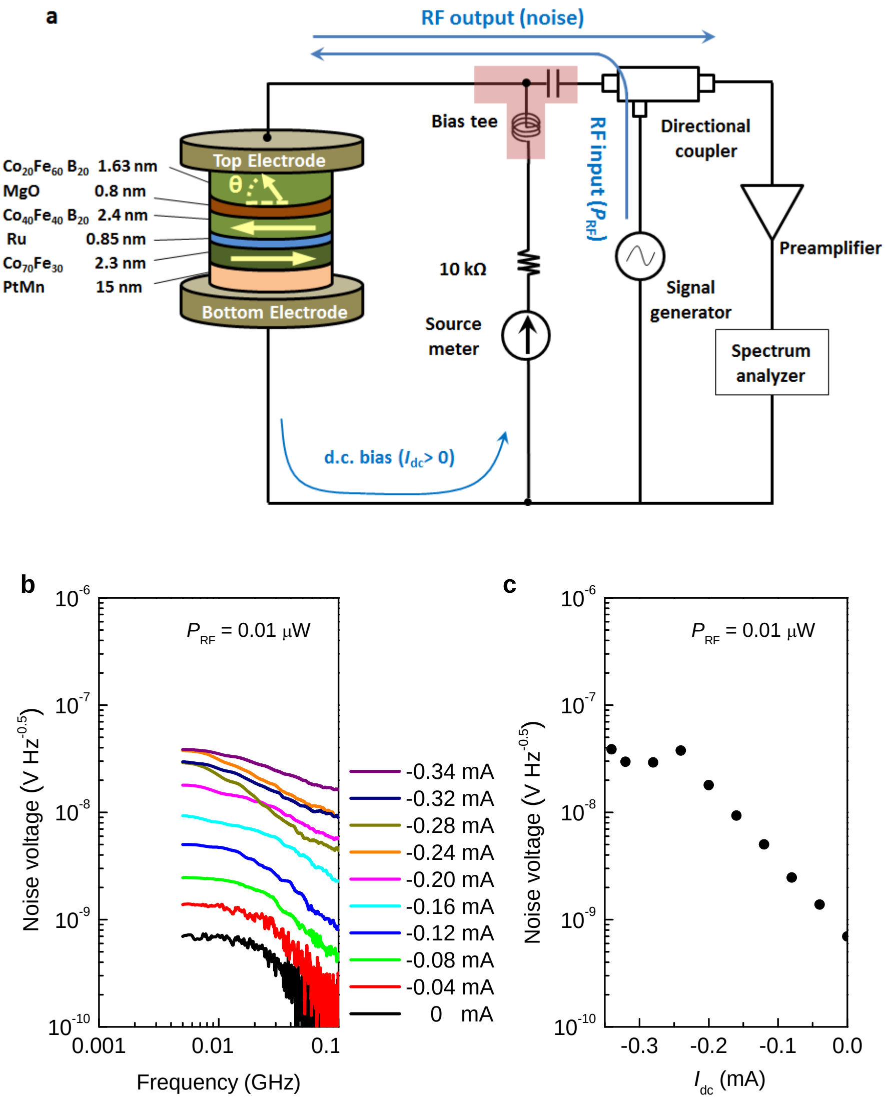

Supplementary Figure 4: Diode-mixing noise. (**a**) The measurement circuit for the diode-mixing noise. (**b**) Noise voltage versus frequency curves for various d.c. bias currents. (**c**) d.c. bias (I_{dc}) dependence of the diode-mixing noise around 0 Hz.

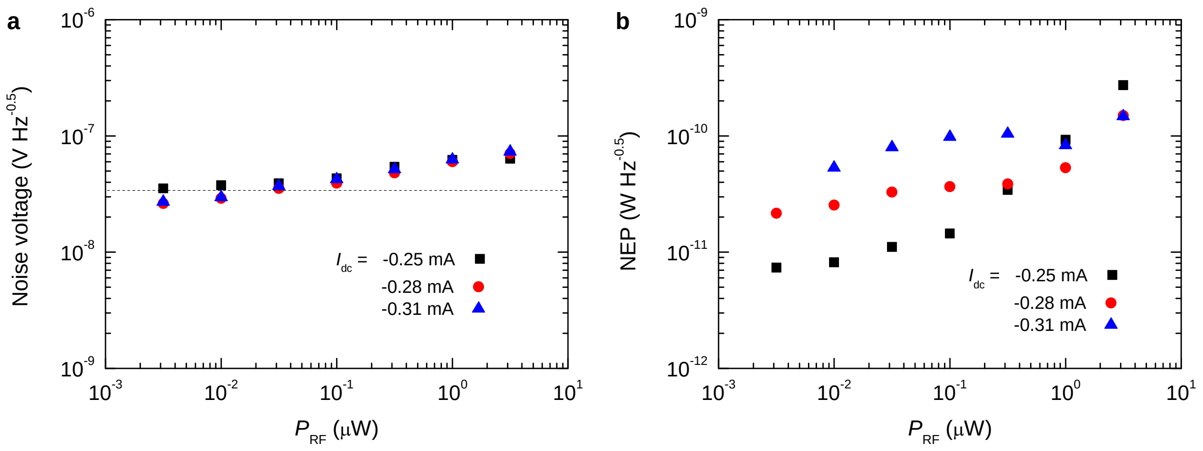

Supplementary Figure 5: Noise equivalent power (NEP). (**a**, **b**) The noise voltage and NEP as a function of RF input power. The points (squares, circles, triangles) are the noise measured with spectrum analyzer, and the dashed line in panel **a** represents the values of nonlinear magnetic noise at I_{dc} = -0.25 mA.

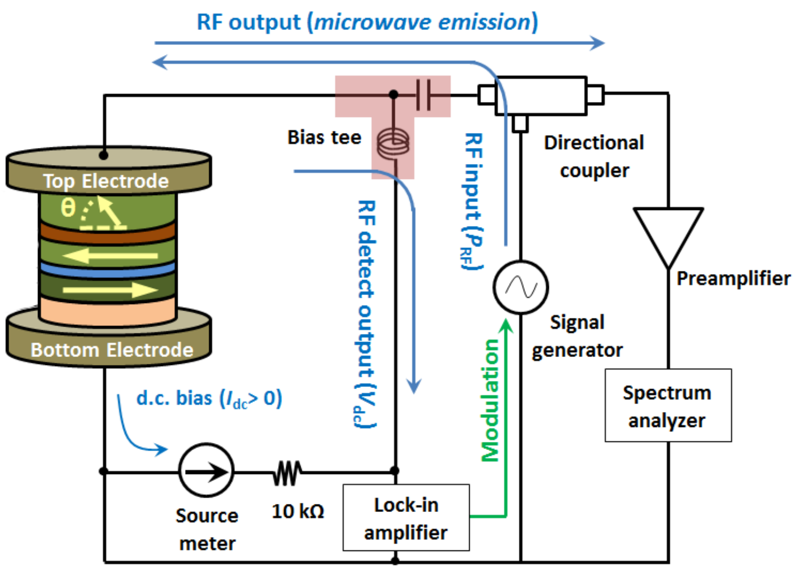

Supplementary Figure 6: The measurement circuit for injection locking experiments. The RF input is applied by a signal generator, the spin-torque microwave emission voltage through the pre-amplifier is measured by a spectrum analyzer, and the spin-torque diode output is simultaneously recorded by a lock-in amplifier.

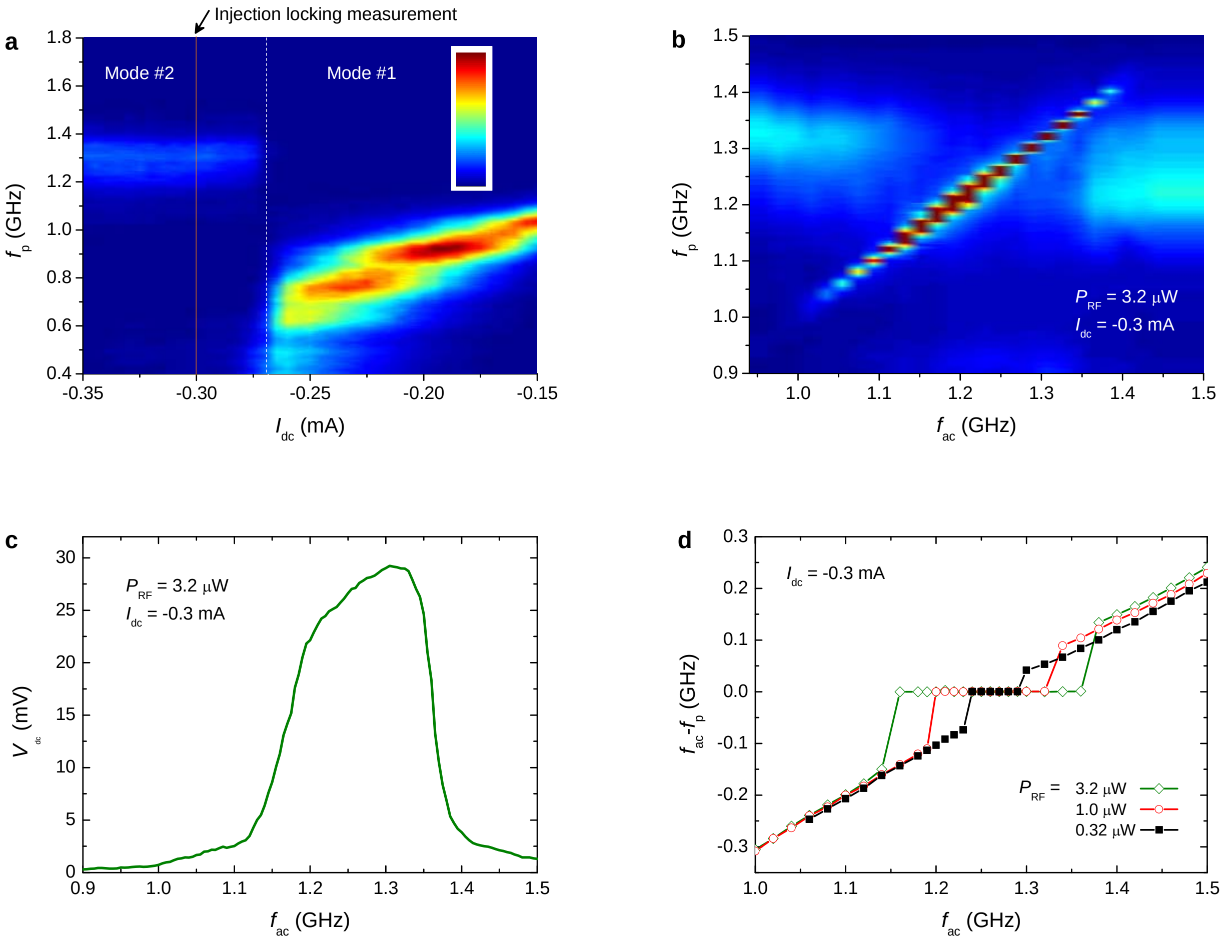

Supplementary Figure 7: Injection locking of spin-torque microwave emission to an external microwave signal for Device #2. (**a**) Plot of spin-torque microwave frequency vs I_{dc}, with amplitude shown in a linear color scale from 0（blue) to 35 nWGHz^{-1} (red). (**b**) Frequency of the microwave emission as a function of the frequency of the external microwave signal source f_{ac}, at RF input power P_{RF} = 3.2 μW. (**c**) Plot of the spin-diode output voltage (V_{dc}). (**d**) The frequency mismatch f_{ac} - f_p as a function of the driving frequency f_{ac} for different RF input powers.

Supplementary Note 1. Estimation of the voltage-controlled perpendicular magnetic anisotropy (VCMA)

The VCMA, i.e. the change of magnetic anisotropy induced by electric field in our devices may increase the sensitivity of the microwave detector. The amplitude of VCMA can be estimated using the method described in Supplementary Ref.[1]. The effective perpendicular magnetic anisotropy energy per unit volume of the free layer, E_p, including interface and demagnetization contributions, can be calculated from the area under the in-plane field-magnetoresistance hysteresis loop, whose shape is identical to that of the magnetic tunnel junction (MTJ) conductance $G(H_x)$ hysteresis loop as shown in Supplementary Fig. 1a

$$E_\mathrm{p} = \int_0^{M_s} H_x(M_x)dM_x = \frac{1}{2}M_\mathrm{s}H_\mathrm{p}, \tag{1}$$

where M_s is the saturation magnetization of the free layer, M_x is the projection of magnetization onto the x axis, and H_p is the effective perpendicular anisotropy field. The VCMA induces a change in the slopes of the two $G(H_x)$ curves in Supplementary Fig. 1a. Supplementary Figure 1b shows the dependence of the perpendicular magnetic anisotropy field on the applied voltage, where $\Delta H_\mathrm{p}(V_\mathrm{dc}) = H_\mathrm{p}(V_\mathrm{dc})-H_\mathrm{p}(0)$. This dependence is well fit by a straight line with a slope of 0.55 kOe V^{-1}, which corresponds to a change in the magnetic anisotropy energy per unit area per applied electric field of 34 fJ $V^{-1}m^{-1}$. This is comparable to VCMA values previously observed in similar systems (Supplementary Refs[1-3]).

Supplementary Note 2. Noise characterization and signal-to-noise ratio

In magnetic tunnel junctions (MTJs) used as radiofrequency (RF) detectors, four types of noise are found, namely Johnson noise, shot noise, nonlinear magnetic noise, and diode-mixing noise. In the device discussed here, the Johnson and shot noise are found to be small compared to the mixing noise and nonlinear magnetic noise, which is consistent with the previous study by Miwa *et al* (Supplementary Ref.[4]). Thus the characterization of both the nonlinear magnetic noise and the diode-mixing noise become important. In this section, we present the noise measurements (the bandwidth is 100 kHz) for the device presented in the main text, and discuss the signal-to-noise ratio (SNR) through the characterization of noise-equivalent power (NEP) (Supplementary Ref.[4]).

a) Nonlinear magnetic noise

The nonlinear magnetic noise is attributed to the nonlinear response of thermal fluctuations in the magnetic free layer. Supplementary Figure 3a shows the measurement circuit for the nonlinear magnetic noise. A d.c. bias is applied to the MTJs by a source meter, and the noise is measured by a spectrum analyzer. Supplementary Figure 3b shows the nonlinear magnetic noise voltage as a function of frequency. The d.c. bias dependence of the peak value around 0 Hz is shown in Supplementary Fig. 3c. The magnetic noise level increases with increasing the d.c. bias

current. Its slight decrease at I_{dc} = -0.28 mA compared to I_{dc} = -0.24 mA is due to a change in the orientation between the free and reference layer magnetizations, which is indicated from the change in the resistance curve (see Fig. 5d in the main text). Our measurements show that this noise is independent of the RF input power, as was the case for the nonlinear magnetic noise reported in Supplementary Ref.[4].

b) Diode-mixing noise

The diode-mixing noise is attributed to the mixing of the input RF current and resistance fluctuations, which originate from thermal fluctuations in the magnetic free layer (Supplementary Ref.[4]). Supplementary Figure 4a shows the measurement circuit for the diode-mixing noise. An RF input at the resonant frequency ($f_{ac} = f_0$) is applied by a signal generator, and the noise voltage through the pre-amplifier is measured by a spectrum analyzer. Supplementary Figure 4b shows the diode-mixing noise voltage as a function of frequency. The d.c. bias dependence of the peak values around 0 Hz is shown in Supplementary Fig. 4c. The mixing noise level exhibits a similar trend as the nonlinear magnetic noise shown in Supplementary Fig. 3c.

c) Noise equivalent power (NEP) in MTJs

Supplementary Figure 5a shows the measured diode-mixing noise around 0 Hz as a function of the RF input power. It can be seen that the nonlinear magnetic noise governs the noise properties when a small RF input power (P_{RF} <0.01 μW) is applied. On the other hand, for large RF input power, the diode-mixing noise becomes more important and proportional to the RF input power. Similar noise properties in MTJ didoes have been observed in Supplementary Ref.[4].

The NEP is characterized to evaluate the signal-to-noise ratio (SNR) in the MTJ. As demonstrated in Supplementary Ref.[4], the NEP in a spin-torque diode is expressed as

$$\mathrm{NEP} = \frac{\text{Noise voltage}}{\text{Sensitivity}} = \frac{V_{\mathrm{noise}}}{\frac{V_{\mathrm{dc}}}{P_{\mathrm{RF}}}}, \quad (2)$$

where V_{dc} is the measured d.c. voltage, P_{RF} is the incident RF power, and V_{noise} is the noise generated in the MTJ device. In our measurement, $V_{noise} = V_{Measured} \times(50+R_{MTJ})/50$, where $V_{Measured}$ is the noise measured with the spectrum analyzer as shown in Supplementary Fig. 5a, and R_{MTJ} is the MTJ resistance (R_{MTJ} = 770 Ω at zero magnetic field). As discussed above and shown in Supplementary Ref.[4], the diode-mixing noise is dominant for a large RF input. Hence, the experimental data of the diode-mixing noise are used to evaluate the RF input power dependence of the SNR. Supplementary Figure 5b displays the NEP values as a function of the RF input for various d.c. currents. As an example, at P_{RF} = 0.01 μW and I_{dc} = -0.25 mA, the measured NEP is estimated to be 8×10^{-12} W Hz$^{-0.5}$, which is of the same order as that in previous MTJ diodes (3.6×10^{-12} W Hz$^{-0.5}$ in Supplementary Ref.[4]).

Supplementary Note 3. Experimental data on an additional device

We found that the detection sensitivity is significantly enhanced for a range of currents, which our simulations link to the presence of the injection locking mechanism. In order to verify this point, we also performed injection locking experiments as shown in Supplementary Figure 6. Our data confirm that the injection locking plays a key role in enhancing the detection sensitivity, as shown in Fig. 5 in the main text.

Here we present additional data from a second device, showing that the injection locking contribution to enhancement of detection sensitivity is robust. The device whose data are presented below is fabricated on the same substrate as the device discussed in the main text. The device has elliptical geometry of 150 nm ×50 nm and a free layer with a thickness of 1.64 nm (Device #2), slightly different from that discussed in the main text. The resistances in parallel and antiparallel configurations are 700 Ω and 1330 Ω, respectively. Supplementary Figure 2a shows the measured rectified voltage as a function of the microwave frequency at I_{dc} = 0 mA for different input microwave powers. The maximum voltage is measured at the resonant frequency f_0 = 1.3 GHz and the detection sensitivity at P_{RF} = 0.01μW was obtained to be 880 mVmW^{-1}, similar to that discussed in the main text. A quadratic dependence (power detection, see Supplementary Fig. 2b) on the RF power is observed as described in Fig. 3a of the main text.

Supplementary Figure 7a shows the microwave emission output as a function of I_{dc} in the absence of RF power. As described in Fig. 5a of the main text, the precession mode changes from Mode #1 to Mode #2, corresponding to the precession axis change in the f_p vs I_{dc} response. The large spin-diode signal (see Supplementary Fig. 7c) and the injection locking behaviors were observed in the Mode #2 region for Device #2. Supplementary Figure 7b shows the microwave output for the oscillation f_p versus the driving frequency f_{ac} at P_{RF} = 3.2 μW. It is clear from Supplementary Fig. 7b that the self-oscillation frequency f_p follows the driving frequency f_{ac} near $f_{ac} = f_p$, similar to that in Supplementary Ref.[5]. Note that the red dots at $f_{ac} = f_p$ are an artifact of the measurement and are due to the signal of the driving source, which cannot be suppressed. When the amplitude of the driving signal increases, the locking range increases (Supplementary Fig. 7d), while the linewidth of the oscillator decreases inside the injection-locking range. At P_{RF} = 3.2 μW, the injection locking range reaches to 0.2 GHz while the oscillation linewidth decreases to about 10 MHz. This corresponds to an improvement by a factor of 15 as compared to the linewidth of the oscillator with no RF power, but is still much larger than the linewidth of the microwave driving signal (which is on the order of several hertz). This behavior can be attributed to the influence of noise (Supplementary Ref.[6]), consistent with a previous study (Supplementary Ref.[7]). If the linewidth of the oscillator is further improved, a larger detection voltage output may be achieved.

Supplementary References

[1] Zhu, J. *et al*. Voltage-induced ferromagnetic resonance in magnetic tunnel junctions. *Phys. Rev. Lett.* **108**, 197203 (2012).

[2] Nozaki T. *et al.* Electric-field-induced ferromagnetic resonance excitation in an ultrathin ferromagnetic metal layer. *Nat. Phys.* **8**, 491-496 (2012).

[3] Alzate, J. G. *et al.* Temperature dependence of the voltage-controlled perpendicular anisotropy in nanoscale MgO/CoFeB/Ta magnetic tunnel junctions. *Appl. Phys. Lett.* **104**, 112410 (2014).

[4] Miwa, S. *et al.* Highly sensitive nanoscale spin-torque diode. *Nat. Mater.* **13**, 50–56 (2014).

[5] Rippard, W., Pufall, M., Kaka, S., Silva, T., Russek, S. & Katine, J. Injection locking and phase control of spin transfer nano-oscillators. *Phys. Rev. Lett.* **95**, 067203 (2005).

[6] Georges, B. *et al.* Coupling efficiency for phase locking of a spin transfer nano-oscillator to a microwave current, *Phys. Rev. Lett.* **101**, 017201 (2008).

[7] Quinsat, M. *et al.* Injection locking of tunnel junction oscillators to a microwave current. *Appl. Phys. Lett.* **98**, 182503 (2011).
